# Devil or angel: A review of autophagy in ischemia–reperfusion injury

**DOI:** 10.1097/MD.0000000000045330

**Published:** 2025-10-17

**Authors:** Guilin Zhou, Lanlan Zhang, Wenya Bai, Jia Liu, Junjie Li, Huan Jiang, Xin Li, Jianlin Shao

**Affiliations:** a Department of Anesthesiology, First Affiliated Hospital of Kunming Medical University, Kunming, China; b Department of Oral and Maxillofacial Surgery, Affiliated Stomatology Hospital of Kunming Medical University, Kunming, China; c Experimental Animal Center, Kunming Medical University, Kunming, China.

**Keywords:** autophagy, cell death, ischemia–reperfusion injury, lysosome, oxidative stress, reactive oxygen species

## Abstract

Ischemia–reperfusion injury (IRI) is a secondary injury that occurs after recanalization of the blood flow in ischemic tissues or organs. Autophagy is a lysosome-dependent cellular process that eliminates misfolded proteins and functionally impaired organelles to maintain intracellular homeostasis. Autophagy plays a pivotal role in IRI occurrence and development. Autophagy acts as a “double-edged sword” in this context, and its role (positive or negative) in IRI remains controversial, complicating efforts to target autophagy to alleviate IRI. In this review, we explore the role of autophagy in various IRI diseases with the aim of providing insights for research focused on mitigating IRI through autophagy regulation.

## 1. Introduction

**Figure 1.**
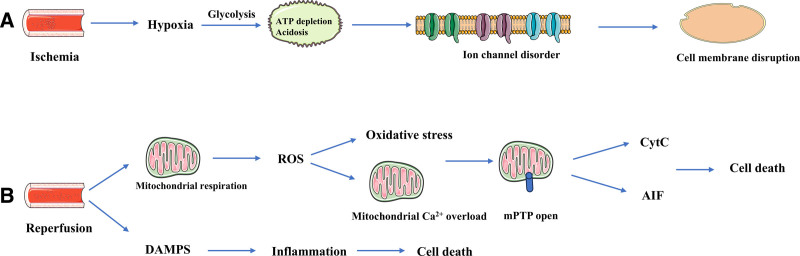
Pathophysiology of ischemia–reperfusion injury. (A) In the ischemic phase, energy deprivation results in disrupted ion exchange, leading to intracellular acidosis and Ca^2+^ overload. Which in turn precipitates mitochondrial damage and the opening of the mitochondrial permeability transition pore, culminating in the release of cell death factors that induce cell death. (B) During the reperfusion phase, blood flow restoration introduces numerous DAMPs, initiating the inflammatory response. Meanwhile, damaged mitochondria generate an abundance of ROS, resulting in oxidative stress that exacerbates cellular damage and leads to cell death. AIF = apoptosis-inducing factor, CytC = cytochrome C, DAMPs = damage-associated patterns, ROS = reactive oxygen species.

Earlier research has focused on minimizing the hypoperfusion duration to alleviate organ or tissue damage. However, functional recovery after recanalization in ischemic areas often falls short of the ideal. Subsequent research has revealed that the restoration of blood flow to ischemic tissues frequently results in more severe secondary injury, termed ischemia–reperfusion injury (IRI).^[1–3]^ IRI comprises both ischemic injury and the ensuing reperfusion injury following blood flow restoration. During the ischemic phase, oxygen is rapidly depleted, leading to the cessation of mitochondrial respiration. Anaerobic metabolism is activated within seconds of the cessation of blood flow, causing intracellular acidosis. ATP produced by glycolysis is insufficient to sustain the ion pump, and the cell begins to swell, causing damage to the cytoplasmic membrane and providing the basis for subsequent more severe injury. During reperfusion, many damage-associated molecular patterns accumulate in large quantities with blood flow, triggering an inflammatory response, and overactivation of inflammation can induce inflammatory cell death, such as apoptosis and pyroptosis.^[4,5]^ At the same time, mitochondrial respiration is recommended as the oxygen supply is reintroduced. The mitochondrial substrate succinate, which accumulates during ischemia, is a powerful source of electrons, generating oxygen free radicals via reverse electron transport through complex I, leading to oxidative stress. ATP is necessary for restoring ionic homeostasis, but it also reactivates calcium transport via the sarcoplasmic reticulum. Excessive Ca^2+^ release from the sarcoplasmic reticulum can lead to mitochondrial calcium overload and the opening of the mitochondrial membrane permeability transition pore (mPTP). With the opening of the mPTP, cytochrome c and apoptosis-inducing factors are released, causing massive cellular damage and death through mechanisms such as apoptosis, necrosis, pyroptosis and autophagy.^[6–8]^ (Fig. 1). Advances in medical technology and the rising prevalence of organ salvage surgeries have implicated IRI in the pathological processes of various diseases (e.g., postrevascularization of stroke and myocardial infarction and liver and kidney transplantations), significantly affecting disease prognosis. Alleviating IRI and improving patient prognosis have thus become primary goals for numerous researchers. Research indicates that various modes of cell death, such as apoptosis, necrosis, pyroptosis, and autophagy, are implicated in IRI and influence the functional outcomes of organs and tissues.^[9–11]^

Autophagy is a lysosome-dependent cellular process that eliminates functionally impaired organelles and misfolded proteins, thereby maintaining intracellular homeostasis. As an intracellular quality control mechanism, autophagy plays a crucial role in IRI across various organs, including the brain, heart, liver, and kidney. Under conditions of IRI or oxygen-glucose deprivation reoxygenation (OGD/R), autophagy frequently plays a dual role in cell survival, akin to a double-edged sword. Intriguingly, even within the same IRI model, autophagy can manifest opposing roles.^[12,13]^ However, identifying factors contributing to this shift in the role of autophagy is challenging.

This review systematically summarizes the literature related to autophagy and IRI over the past 5 years, aiming to identify the factors influencing this ambivalent effect of autophagy in IRI and to offer insights into mitigating IRI by strategically targeting the regulation of autophagic pathways.

## 2. General overview of autophagy

**Figure 2.**
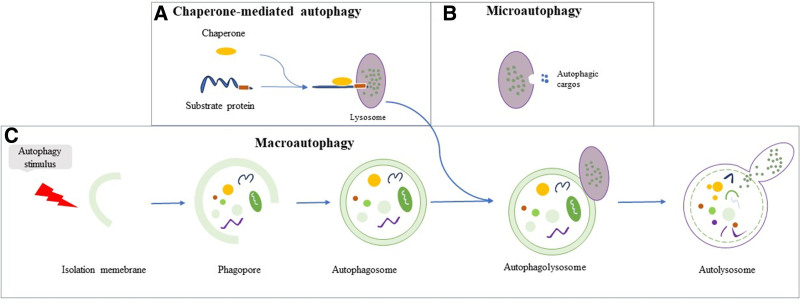
Autophagy process overview. (A) Chaperone-mediated autophagy: proteins with specific motifs in the cytosol are identified by molecular chaperones and conveyed to the lysosomal membrane. Here, they bind to CMA receptors, undergo internalization, and are then degraded by lysosomal hydrolases. (B) Microautophagy: the lysosomal membrane invaginates, enveloping and internalizing the autophagic substrate. This is then hydrolyzed by lysosomal hydrolases. (C) Macroautophagy: cytoplasmic components are encapsulated by bilayer membranes, originating from the nonribosomal regions of the endoplasmic reticulum, the Golgi apparatus, among others. These components are then encapsulated in bilayer membrane vesicles regulated by ATGs. Following this, the vesicles fuse with lysosomes, where lysosomal hydrolases degrade the encapsulated substrates. ATGs = autophagy-related genes, CMA = chaperone-mediated autophagy.

The concept of autophagy was first introduced in 1963 by Christian René de Duve.^[14]^ Subsequently, Yoshinori Ohsumi, a Japanese scientist, initially elucidated the morphology and molecular mechanisms of autophagy in yeast cells and identified a series of autophagy-related genes (ATGs).^[15,16]^ Since then, extensive research on autophagy has led to a more comprehensive understanding of its processes and mechanisms, thereby enhancing our knowledge of the pathological processes of various diseases. As a complex intracellular process, autophagy degrades dysfunctional cellular components via the lysosomes. Regulated by ATGs, eukaryotic cells utilize lysosomes to eliminate misfolded or damaged organelles and proteins, thereby maintaining intracellular energy balance.^[17]^ Autophagy is categorized into macroautophagy, microautophagy, and chaperone-mediated autophagy (CMA), differentiated by the substrate type, transport mode, and regulatory mechanisms. Macroautophagy (subsequently referred to as autophagy) is initiated in response to cellular stress. Initially, the cytoplasm is enveloped by a bilayer membrane, originating from the nonribosomal regions of the endoplasmic reticulum (ER) and Golgi apparatus, among others. The autophagosome encapsulates the targeted substrate for degradation and subsequently fuses with the lysosomes. Lysosomal hydrolases release substrates within the vesicles, and the small molecules produced through hydrolysis are subsequently released into the cytoplasm via the lysosomal membrane for cellular reuse^[18,19]^ (Fig. 2). In microautophagy, a similar encapsulation process occurs wherein the substrate is enveloped by the invaginating lysosomal membrane^[20]^ (Fig. 2). CMA involves proteins with a specific motif (KFERQ) in the cytoplasm being recognized by molecular chaperones, transported to the lysosomal membrane, and subsequently binding to it, internalizing via the CMA receptor, and degradation by lysosomal enzymes^[21]^ (Fig. 2). The substrates of autophagy are diverse, including misfolded protein aggregates, oxidized lipids, pathogens, and damaged intracellular organelles, such as mitochondria, which can adversely affect cellular health if not adequately removed. Autophagic degradation products are recycled to fulfill the cell’s nutritional requirements for energy production and serve as a basis for the biosynthesis of new cellular components that are crucial for maintaining intracellular homeostasis.^[22]^

Dysregulated autophagy has been implicated in numerous pathological processes, including IRI,^[23]^ cancer, aging, cardiovascular disease, and liver and kidney disease.^[24]^

## 3. Autophagy in IRI

Autophagy is involved in the pathology of IRI in a wide range of organs.^[25–27]^ The following focus on the role that autophagy plays in common IRI diseases.

### 3.1. Autophagy in cerebral IRI

Stroke is the second leading cause of death globally, accounting for 11.6% of the total mortality. Furthermore, ischemic stroke constitutes over half of all stroke-related fatalities.^[28]^ The primary treatment objective for ischemic stroke is prompt restoration of blood flow and oxygen supply to the ischemic region. However, restoring blood flow to the ischemic brain can exacerbate functional brain injury, a condition known as cerebral ischemia–reperfusion injury (CIRI).^[29]^ Regulation of autophagy is crucial for cell survival following ischemic stroke. Although appropriate autophagy exerts a protective effect on ischemic neural tissues, excessive autophagy can result in cell death.^[30,31]^

Electroacupuncture (EA), a contemporary acupuncture technique, integrates needle insertion with pulsed electric currents to stimulate the acupuncture points. EA has shown potential benefits in the treatment of ischemic stroke.^[32]^ Research in rats indicates that EA pretreatment can inhibit autophagy through the activation of the SIRT1-FOXO1 signaling pathway, offering neuroprotective effects against CIRI.^[33]^ Hou et al^[34]^ developed an OGD/R model using PC12 cells to simulate CIRI in vitro and explored the underlying mechanism. These findings indicate that curcumin alleviates neuronal IRI by inhibiting hypoxia-induced factor-1α (HIF-1α) and autophagy. Melatonin (MT) is protective against CIRI, though its regulatory mechanisms remain to be elucidated. Subsequent experiments revealed that MT intervention significantly reduced LC3 II/LC3 I ratios, increased p62 levels, improved neurological function scores, and decreased neuronal apoptosis rates post-CIRI in rats. Further investigations have shown that MT’s protective effects of MT are mediated by regulating the miR-26a-5p-NRSF axis and JAK2-STAT3 pathway, thereby reducing autophagy levels during CIRI.^[35]^ Ischemic postconditioning (IPostC) is a short period of mild, nonfatal ischemia in the early stages of CIRI. To confirm whether IPostC plays an important role in CIRI by downregulating miR-124 expression, Miao et al^[36]^ treated mice with brain I/R and IPostC based on MCAO. The results showed that IPostC significantly reduced the neurobehavioral deficits and cerebral infarct volume. In addition, inhibition of miR-124 effectively reduced neuronal apoptosis in vivo and in vitro and activated the PI3K/Akt/mTOR signaling pathway, thereby inhibiting apoptosis and autophagy. Mitochondria-associated ER membranes (MAMs) serve as crucial bridges connecting the ER and mitochondria within cells. Vesicle-associated membrane protein-associated protein B (VAPB) and protein tyrosine phosphatase interacting protein 51 (PTPIP51) are responsible for the formation and stability of MAMs and have been implicated in the pathogenesis of various diseases.^[37]^ After MACO/R, MAMs were impaired and VAPB-PTPIP51 expression was reduced in rat brain tissue. After VAPB or PTPIP51 knockdown, the damage to MAMs is exacerbated, accompanied by excessive autophagy activation and increased ration of substantial production, leading to increased infarct size and exacerbated neurological deficits.^[38]^

While many studies suggest that autophagy acts as a disruptor in CIRI and that enhanced autophagy may exacerbate CIRI, other studies present contrasting findings. miR-187-3p has been implicated in CIRI. Inhibition of miR-187-3p expression can enhance autophagic flux via seipin modulation, offering protection against OGD/R-induced apoptosis in PC12 cells.^[39]^ Astragaloside IV (AIV) has demonstrated potential as a neuroprotective agent. Studies on its mechanism suggest that AIV may exert neuroprotective effects by enhancing autophagy and reducing apoptosis.^[40]^ The inhalation anesthetic isoflurane (ISO), widely used in clinical anesthesia, plays an important protective role against central nervous system injury.^[41]^ A study in the rat MCAO/R model found that ISO-treated MCAO rats had significantly lower neurobehavioral scores, significantly higher expression of AMPK, ULK-1, Beclin-1, and LC3B, and significant improvement in cognitive memory function. Inhibition of the autophagy pathway or AMPK, a key protein in autophagy, resulted in significantly higher neurobehavioral scores and protein expression of NLRP3, IL-1β, and IL-18 in rats.^[42]^ An enriched environment (EE) is an effective rehabilitation intervention, and EE exposure has been shown to protect against adverse effects associated with cerebral IRI.^[43]^ Zhang et al^[44]^ exposed transient middle cerebral artery occlusion (tMCAO) mice to EE or standard conditions for 7 days and then studied their neurological deficits, autophagy- and inflammation-related proteins, and mitochondrial morphology and function. EE was found to enhance autophagic flux by inhibiting mTOR and enhancing mitochondrial autophagic flux through the recruitment of Drp1 and Parkin, resulting in the elimination of dysfunctional mitochondria, thereby suppressing inflammation and alleviating neurological deficits. Lin28 is a highly conserved RNA-binding protein, and Lin28a is downregulated in CIRI animal models and OGD/R-induced neuronal cells. Upregulation of Lin28a reduces cerebral infarction induced by I/R, attenuates neurological damage, and reduces neuronal apoptosis by regulating Sirt3-induced autophagy.^[45]^

The results concerning CIRI have pointed out that for rats and mice, the difference in anesthetics used in the modeling process has a significant effect on autophagy; autophagy becomes milder and more angel-like, and activation of autophagy attenuates CIRI in animals modeled with anesthetics with analgesic effects such as pentobarbital and isoflurane (ISO), as compared to chloral hydrate anesthesia.^[33,36,42,45]^ When there is a difference in body weight of the rats, it seems that the larger the body weight, the more moderate the autophagy after modeling.^[35,40]^ In a mouse model of CIRI, when the duration of ischemia was determined, the role of autophagy gradually changed from that of a devil to that of an angel as the duration of reperfusion was prolonged.^[38,44,45]^ For cells, shorter H/R times induce milder autophagy,^[34,39]^ and different cell types also affect the level of autophagy,^[34,40]^ perhaps because of the inconsistent tolerance of different cells to hypoxia.

### 3.2. Autophagy in myocardial IRI

According to the World Health Organization (WHO), ischemic heart disease is projected to remain the world’s leading cause of death by 2022, comprising 16% of all global fatalities.^[46]^ Primary treatments for ischemic heart disease include percutaneous coronary intervention, coronary artery bypass grafting and thrombolysis. Timely reperfusion of ischemic myocardium is crucial for rescue. However, this reperfusion can aggravate myocardial injury, a phenomenon known as myocardial ischemia–reperfusion injury (MIRI).^[47]^ Autophagy, an intracellular quality control system, plays a role in the development of MIRI, a complex intracellular process. However, the role of autophagy in MIRI remains to be elucidated.

Resveratrol (RES), a natural polyphenolic compound found in red wine and grape skins, has attracted significant attention due to its cardioprotective properties.^[48]^ A study investigating the effects of RES on MIRI by ligating the left anterior descending branch of rats and subjecting H9c2 cells to hypoxia/reoxygenation (H/R) found that RES attenuated MIRI by inhibiting autophagy through DJ-1 regulation of the MEKK1/JNK pathway. PCSK9 is an amino acid serine protease encoded by the PCSK9 gene on human chromosome 1 p32.3, mainly expressed in hepatocytes.^[49]^ Knockdown of PCSK9 inhibits the Bnip3-mediated autophagy pathway, resulting in alleviation of MIRI and improvement in inflammatory response, myocardial infarct size, and cardiac function.^[50]^ Epigallocatechin-3-gallate (EGCG), an active polyphenol flavonoid or catechin, exerts bioactivity in tissues with various diseases and protects the ischemic myocardium.^[51]^ A recent study found that EGCG pretreatment exerts cardioprotective effects through 14-3-3η inhibition of MIRI-induced ferroptosis, autophagy and apoptosis.^[52]^ Collectively, these studies indicate that autophagy exacerbates I/R-induced myocardial injury and that mitigating autophagy levels can protect the myocardium from IRI.

However, other studies have shown contrasting results, indicating that enhancing autophagy in MIRI using various therapeutic approaches can attenuate MIRI. Transient receptor potential mucolipin 1 (TRPML1), encoded by *MCOLN1*, is a nonselective cation channel that transports Ca^2+^, Fe^2+^, and Zn^2+^.^[53]^ Inhibition of TRPML1 attenuates MIRI via a mechanism that may be mediated by enhanced autophagic flux in cardiomyocytes.^[54]^ Shexiang Baoxin Pill (SBP), a renowned traditional Chinese herbal formula, is commonly used to treat cardiovascular diseases. It has been discovered that SBP activates autophagy to mitigate MIRI via the Cerna-Map3k8 pathway.^[55]^ Galangaloside (Gal), a natural flavonoid derived from Chinese herbs, improves cardiac function and attenuates MIRI by promoting autophagy and anti-inflammatory effects.^[56]^ Myocardin-related transcription factor A (MRTF-A) is a transcriptional modulator widely expressed in the cardiovascular system. To examine the role of MRTF-A in MIRI at the whole-animal level, Zhong et al^[57]^ found that overexpression of MRTF-A significantly reduced NLRP3 inflammasome activity and increased the expression of autophagy proteins in myocardial ischemic tissues to attenuate MIRI, as verified by the establishment of a rat model of MIRI in conjunction with histological, morphological, and functional experiments. Cordycepin is a major bioactive component extracted from the traditional Chinese herb, Cordyceps sinensis. A recent in vivo and in vivo experimental study found that cordycepin enhanced autophagy through the AMPK-mTOR signaling pathway, exerted cardioprotective effects, and promoted the recovery of cardiac function after MIRI.^[58]^ Taohong Siwu decoction (TSD) is a classic traditional Chinese medicine (TCM) prescription used to promote the blood circulation and alleviate blood stasis. Yang et al^[59]^ conducted in *vivo* experiments in mice to investigate the effect of TSD on MIRI and explore the underlying mechanisms. They discovered that TSD significantly increased the number of autophagosomes in myocardial tissue and TSD administration increased the protein expression of microtubule-associated protein light chain3 (LC3), and reduced the protein expression of p62. However, the cardioprotective effect of TSD was reversed by 3-Methyladenin. ZNF143 is an important transcription factor that binds to the promoters of related genes. Raptor is a key component of the mTORC1 complex, which regulates autophagy. Overexpression of ZNF143 exacerbates MIRI by upregulating Raptor expression and reducing autophagic activity.^[60]^ Resveratrol (RSV) is a plant-derived polyphenol compound that has been shown to reduce oxidative stress in cardiomyocytes by activating the AMPK/SIRT1-FOXO1 signaling pathway and enhancing autophagy levels, thus alleviating MIRI.^[61]^

In studies in which the experimental animals were mice in MIRI, an ischemia time of 1 hour appeared to be a watershed, and when myocardial ischemia time was <1 hour, autophagy behaved relatively modestly and exerted a protective effect on the myocardium after MIRI for both short reperfusion times (e.g., 2h or 4h)^[55,58,59,61]^ and relatively longer reperfusion times (e.g., 24 hours).^[54,56]^ However, when the ischemia time reached 1h, autophagic flux appeared to be overactivated, adversely affecting ischemic myocardial tissue.^[51]^ Intriguingly, in studies using the rat model of MIRI, very different results from those in mice were presented. When myocardial ischemia was limited to 30 minutes, autophagy played a pivotal role in both short (e.g., 2 hours)^[62]^ and prolonged reperfusion (e.g.,3d).^[50]^ When the ischemic time was prolonged (e.g., 40 minutes or 45 minutes),^[57,60]^ autophagy levels became mild again and became an angle of the myocardial tissue. We also observed that the weekly age and body weight of rats and mice did not seem to affect the shift in the role of autophagy during MIRI.

In cellular experiments, the sensitivity of different cell types to hypoxia and reoxygenation is markedly different, and the role of autophagy remains elusive in different types of cell models. In H9c2 cells, when hypoxia and reoxygenation times were the same, low oxygen concentration (1% O_2_) and complete absence of oxygen during oxygen-glucose deprivation caused equal levels of autophagy activation.^[51,62]^ However, when the duration of hypoxia was prolonged from 2h to 3h, a mild level of autophagy was overactivated.^[60,62]^ In contrast, neonatal rat ventricular myocytes are not sensitive to the duration of hypoxia, and the levels of autophagy are moderate for hypoxia durations between 2 and 4 hours.^[54,58,61]^ Moreover, during oxygen-glucose deprivation, low oxygen concentration (1% O_2_)^[61]^ and complete anoxia^[58]^ have no effect on the role shift of autophagy.

### 3.3. Autophagy in renal IRI

Acute kidney injury (AKI), formerly known as acute renal failure, is a pathological condition of the renal system characterized by high morbidity and mortality.^[63]^ IRI is the predominant cause of AKI. Renal ischemia–reperfusion injury (RIRI) is a pathophysiological process exacerbated when kidney tissues that are already damaged by ischemia are re-exposed to blood flow. This situation is further complicated, as ischemia leads to early and irreversible tubular damage, thereby exposing the tissue to further reperfusion injury.^[64,65]^ Autophagy also plays an important role in RIRI development. In RIRI, the autophagic response to cellular stress serves as an adaptive and cytoprotective mechanism.^[66]^ Autophagy mitigates cell death in RIRI by eliminating damaged macromolecules and organelles and positioning it as a potential target for therapeutic intervention. However, it is also posited that autophagy may exacerbate cell death under excessive ischemic conditions.

Autophagy may exacerbate cell death in conditions of excessive ischemia. miR-30a-5p, a miRNA extensively involved in IRI, has been shown to diminish RIRI by inhibiting autophagy and apoptosis via the Beclin-1/ATG16 pathway.^[67]^ ALDH2, a crucial enzyme in mitochondrial ethanol metabolism, oxidizes both intracellular and extracellular aldehydes and plays a role in various pathological processes, including oxidative stress in MIRI and the activation of the autophagy-related protein Akt.^[68,69]^ Based on this, Lin et al^[70]^ hypothesized that ALDH2 might mitigate RIRI by regulating excessive autophagy in HMP, and developed HMP-treated rabbit models with either ALDH2 agonistic or inhibitory autologous kidney transplantation. They confirmed that ALDH2 inhibits autophagy via the Akt-mTOR pathway and reduces the toxicity of 4-hydroxynonenal in HMP, thereby reducing IRI and protecting donor kidneys. The involvement of Na+/K+-ATPase pumps in RIRI has been previously established. Inhibition of Na+/K+-ATPase was found to reduce autophagy levels, improve renal histopathology, and enhance renal function, as indicated by serum urea nitrogen and creatinine levels post-IRI.^[71]^

In contrast, autophagy eliminates damaged macromolecules and organelles, thereby attenuating cell death in RIRI, making it a potential target for therapeutic interventions. Leucine-rich α2-glycoprotein 1 (LRG1) is a pleiotropic leucine-rich repeat sequence protein that has been shown to be involved in the process of CIRI.^[72]^ To investigate the exact role of Lrg1 in RIRI, Chen et al^[73]^ established a mouse RIRI model and an HK-2 cell hypoxia model to explore the nephroprotective mechanism of silencing Lrg1 and found that Lrg1 silencing could serve as a potential therapeutic target for inhibiting the TGFβ1-Smad1/5 pathway, thereby enhancing autophagy and reducing apoptosis in patients with AKI. Trehalose (Tre), a nonreducing disaccharide, has been shown to be therapeutically effective in various diseases as an autophagy enhancer independent of the mammalian target of rapamycin (mTOR). A study exploring whether Tre plays a protective role in RIRI found that Tre ameliorates RIRI by enhancing autophagy-mediated inhibition of inflammatory responses, apoptosis, and oxidative stress.^[74]^ Another study found that ebselen pretreatment enhanced autophagy, activated the Nrf2 pathway, and attenuated oxidative stress to alleviate RIRI.^[75]^ Melatonin (MT) is a methoxyindole hormone synthesized by the pineal gland and its role in circadian rhythm regulation has been widely demonstrated. However, little is known about the effects of MT on cell survival and autophagy in renal IR. A study conducted in female mice to assess the effects of MT pretreatment on IR-induced renal injury found that MT pretreatment ameliorated oxidative stress, attenuated apoptosis, and significantly improved renal function by promoting autophagy through the TLR4/MyD88/MEK/ERK/mTORC1 signaling pathway.^[76]^

The above briefly summarizes the various studies on the role of autophagy in RIRI. These studies showed that in mice, autophagy levels appeared to be more moderate when renal ischemia lasted <45 minutes (e.g., 35 or 40 minutes) and played a significant protective role in RIRI.^[73,74,76]^ Once the renal ischemia time reaches 45 minutes or 1 hour, autophagy is overactivated and adversely affects the kidney regardless of the reperfusion time.^[67,71]^ However, in rats, autophagy plays a protective role, even when renal ischemia reaches 45 minutes.^[75]^ Body weight and age of mice seem to have little effect on autophagy levels after RIRI.

Oxygen-glucose deprivation in HK-2 cells is commonly used to mimic an in vitro RIRI model. However, in the above studies, the role of autophagy in the in vitro model of RIRI established by HK-2 cells was elusive because of the inconsistencies in the modeling approach, the use of medium and the timing of H/R, all of which need to be explored in more relevant studies.^[67,73]^

### 3.4. Autophagy in hepatic IRI

Hepatic ischemia–reperfusion injury (HIRI) is a major complication of liver surgeries, such as liver resection and transplantation, and is a primary cause of liver insufficiency or failure.^[77]^ Ischemic injury causes hypoxia, ATP depletion, pH alterations, and cellular metabolic stress, culminating in initial cellular damage or death.^[78]^ During subsequent reperfusion injury, hepatic metabolism is disturbed, triggering an interrelated inflammatory cascade that further exacerbates hepatocyte injury.^[79]^ Under normal conditions, cells maintain baseline autophagic activity, which is heightened during nutrient deficiency, abnormal energy metabolism, ischemia, hypoxia and pathogen infection. Owing to the mutual association with the induction of nutritional deficiency, a close link exists between HIRI and autophagy.^[80]^ Autophagy, often described as a “double-edged sword” plays an equally ambiguous role in HIRI.

Studies have shown that autophagy may exacerbate HIRI, leading to the emergence of the idea of inhibiting autophagy as a therapeutic strategy to mitigate HIRI. IL-37, a member of the IL-1 family, plays a well-established role in liver diseases.^[81]^ A study exploring the role and mechanism of IL-37 in HIRI observed aberrant autophagy activation in HIRI models both in vivo and in vitro. Further research demonstrated that IL-37 can attenuate HIRI by inhibiting overactive autophagy and apoptosis via the AMPK/mTOR/ULK-1 signaling pathway.^[82]^ Interferon regulatory factor 1 (IRF-1) is a primary member of the IRF family, which is closely associated with the development of many liver diseases.^[83]^ A study on wild-type and IRF-1 knockout mice found that high expression of IRF-1 during HIRI was associated with increased liver injury and that autophagy was activated as shown by autophagy marker proteins and the detection of autophagosomes. These effects were diminished by IRF-1 deficiency in IRF-1 knockout (KO) mice. In addition, the autophagy inhibitor 3-MA showed the same effects as the IRF-1 knockout. This experiment also revealed that β-linker protein expression decreased during liver IR and increased in IRF-1 KO mice. Immunoprecipitation assays showed that IRF-1 bound to β-catenin. Overexpression of IRF-1 induces autophagy and inhibits β-catenin expression. β-catenin inhibitors increase autophagy, whereas β-catenin agonists inhibit autophagy in primary mouse hepatocytes. These results suggest that IRF-1 exacerbates HIRI by inhibiting autophagy activation of β-conjugated proteins in mice.^[84]^

Numerous studies have indicated that autophagy aids hepatic recovery after I/R. HIRI often results in severe hepatic injury and dysfunction, typically linked to disruption of autophagy and the endogenous cannabinoid system. Rezq et al^[85]^ explored the role of rimonabant, a cannabinoid receptor 1 (CB1R) antagonist, in regulating autophagic response after HIR injury using a rat HIRI model. They discovered that rimonabant significantly reduced HIR-induced liver injury, inflammation, fibrosis, and oxidative stress and ameliorated the associated pathological features. Additionally, rimonabant modulated the expression of p62, Beclin-1 and LC3; reduced CB1R levels and diminished the activities of pERK1/2, PI3K, Akt and mTOR. Transient receptor potential Melastatin 2 (TRPM2) is crucial in organ IRI.^[86,87]^ Zhang et al^[88]^ conducted a series of in vivo and in vitro experiments to investigate the role and mechanism of TRPM2 in HIRI. In their in vivo experiments, they used a knockout mouse HIRI model to assess TRPM2’s impact on HIRI. For in vitro experiments, they established a human hepatocyte H/R model and used autophagy agonists, inhibitors, and NLRP3 inhibitors to explore TRPM2’s mechanism of action. Downregulation of TRPM2 has been shown to protect the liver from I/R and H/R injury by activating autophagy and inhibiting the NLRP3 inflammasome pathway. Remote ischemic preconditioning (RIPC) protects target organs from damage caused by reduced blood flow by inducing the release of endogenous substances and cytokines.^[89]^ HIRI damages hepatocytes by activating ER stress-dependent apoptosis, whereas RIPC significantly attenuates hepatic injury and inflammatory cell infiltration by activating autophagy.^[90]^ Polyethylene glycols (PEG) are nontoxic, neutral, water-soluble compounds, PEG35 with a molecular weight of 35kD was shown to ameliorate H/R-induced hepatocyte injury by enhancing autophagy.^[91]^ The Hippo pathway is an evolutionarily conserved signaling cascade that regulates organ size, tissue regeneration, and stem cell self-renewal. The downstream transcriptional co-activator Yes-associated protein (YAP) is a key component of the Hippo pathway.^[92]^ A recent study found that targeting the Hippo (YAP)-JNK-autophagy axis may protect against HIRI by activating autophagy and inhibiting apoptosis in hepatocytes.^[93]^ Bone marrow mesenchymal stem cells (BM-MSCs) can differentiate into hepatocytes in vivo, and hepatocyte exosomes are important for inhibiting apoptosis and promoting hepatocyte regeneration.^[94]^ Yang et al investigated whether MSC-derived exosomes from hepatocyte-like cells (MSC-HEPS-Exos) could mitigate liver IRI. They induced MSC differentiation into hepatocyte-like cells, extracted exosomes, and injected them into a mouse liver I/R model via tail vein. Concurrently, CoCl2 was used to simulate IRI in vitro. They found that, in vivo, MSC-HePS-Exo effectively attenuated HIRI, reduced hepatocyte apoptosis and decreased liver enzyme levels. Consistent with the in vivo results, the in vitro experiments confirmed that MSC-HePS-Exos increased hepatocyte ischemia tolerance and reduced apoptosis. Additionally, enhanced autophagy was observed in both in vivo and in vitro experiments following exosome intervention. This suggests that MSC-HePS-Exos may exert a protective role by enhancing autophagy.^[95]^

The partial (70%) warm IRI model of C57BL/6J mice is predominantly used in HIRI studies. After a comparative summary, we found that in the mouse HIRI model, when the reperfusion time was 6h, the ischemia time was prolonged from 40 minutes to 1.5h, and the autophagy levels were all at a relatively mild level, which was more like an angel to the injured liver.^[88,90,92,95]^ At an ischemia time of 1 hour, the reperfusion time was prolonged from 6 to 12 hours, the initially mild autophagy was then overactivated.^[82,95]^ Differences in anesthetics also seem to affect autophagy role shifts,^[84,95]^ while no effect of weekly mouse age and body weight on autophagy levels was observed.

Unfortunately, due to differences in cell types (AML-12, L-02, HepG2, THLE2), mode of hypoxia (physical and chemical), H/R time, and culture media (DMEM, RMPI 1640), it is very difficult to screen for factors affecting the level of autophagy in the above in vitro experiments.

### 3.5. Autophagy in intestinal IRI

Intestinal ischemia–reperfusion injury (IIRI) is a multifactorial disease triggered by severe trauma, infection, shock resuscitation, intestinal obstruction, among other causes.^[96]^ Autophagy is recognized as a key regulator of IIRI, although its precise role remains debated. miR-146a-5p plays a role in the onset and progression of IRI.^[97]^ Zhenzhen et al^[98]^ developed IIRI models both in vivo and in vitro, discovering that miR-146a-5p inhibits autophagy and attenuates IIRI by targeting TXNIP and modulating the PRKAA/mTOR pathway. Another study revealed that miR-182 upregulation attenuates IIRI by targeting Deptor, inhibiting mTOR activity, and suppressing autophagy.^[99]^ Propofol, an intravenous anesthetic with organ-protective properties, and AMPK, a key cellular energy sensor, are linked to oxidative stress and inflammation. Activation of the AMPK-Sirt1-autophagy signaling pathway occurs after intestinal I/R. Propofol pre-adaptation or posttreatment further activates this pathway, exerting anti-inflammatory, antioxidant, and anti-apoptotic effects, thus attenuating IIRI.^[100]^ IPostC offers significant protection against IIRI. To investigate the mechanism of IPostC, Chen et al^[101]^ developed an intestinal I/R mouse model and conducted a 10-second reperfusion-ischemia treatment, repeated over 3 cycles, to simulate IPostC. Simultaneously, autophagy and pathway inhibitors were administered to examine the role of autophagy and related pathways in this process. This study confirmed that IPostC promotes autophagy and inhibits oxidative stress by activating the Akt/GSK-3β/Nrf2 pathway, thereby alleviating IIRI.

In the IIRI study. In in vivo experiments, when the reperfusion time was kept constant, prolongation of the ischemia time from 45 minutes to 1 hour led to a loss of autophagy protection, which exacerbated tissue damage in animal models.^[98,99,101]^ Another rat study demonstrated different results, in which autophagy still exerted a protective effect on the injured intestine, even when the ischemia time reached 1 hour or 1.5 hours.^[100,102]^ The results described above suggest that the role of autophagy in IIRI studies may be related to the duration of ischemia and animal species, which is consistent with previous studies on autophagy and MIRI.

## 4. Discussion

**Figure 3.**
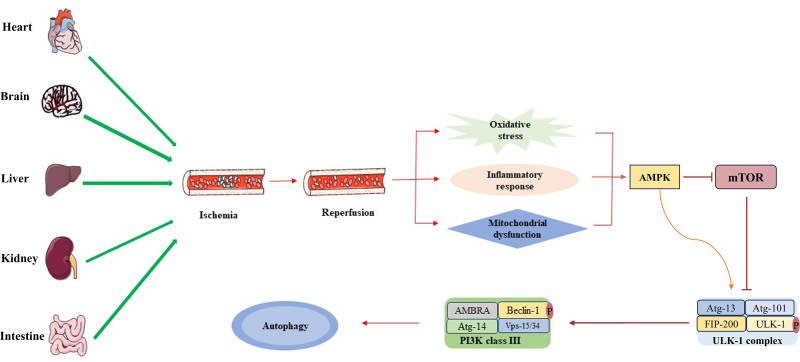
Induction of autophagy in IRI. During IRI, organs experience insufficient energy supply, leading to mitochondrial dysfunction and the generation of substantial ROS, which in turn cause oxidative stress. This stress is further compounded by the release of a large number of DAMPs, triggering inflammatory responses. In response, AMPK is activated while mTOR is inactivated. The activation of AMPK facilitates the phosphorylation of ULK-1, subsequently activating the ULK-1 complex.^[103]^ This activated ULK-1 complex associates with phagocytic vesicles and forms ULK-1 dots on their membranes. These dots recruit the Vps34-like complex and phosphorylate Beclin-1 within the complex, enhancing the activity of the Vps34 complex. This process promotes the induction and maturation of autophagy.^[104]^ AMPK = AMP-activated protein kinase, DAMPs = damage-associated patterns, mTOR = mammalian target of rapamycin, ROS = reactive oxygen species, ULK-1 = Unc-51 like autophagy activating kinase 1.

Autophagy plays a dual role in pathophysiological processes, either by promoting cell survival or contributing to cell death. This review summarizes the role of autophagy across various types of IRI, revealing its occurrence in diverse target organ IRI models (Fig. 3). During the process of organ IRI, mitochondrial dysfunction caused by insufficient energy supply leads to the release of large amounts of ration of substantial and damage-associated molecular patterns, thereby triggering oxidative stress and inflammatory responses. In response to this injury, the AMPK-ULK-1 signaling pathway is activated, leading to the formation of the ULK complex.^[103]^ Subsequently, this complex localizes to the phagophore membrane, recruits the Vps34-like complex, induces the phosphorylation of Beclin-1, and activates VPS34 lipid kinase to initiate autophagy.^[104]^ In some instances, autophagy mitigates IRI, whereas in others, it exacerbates the condition. The dynamic nature of autophagy’s role is suggested to be related to the extent and duration of autophagy activation as well as the specific cellular or animal disease context,^[105]^ aligning with our own conclusions. Nonetheless, the link between these differences in cells, target organs, and animal models and autophagic activity is yet to be fully elucidated, necessitating further comprehensive studies.

Analyzing the above studies of autophagy in IRI in different organs, we found that in the study of CIRI, the choice of anesthetics (chloral hydrate, pentobarbital and ISO), the weight of the animal, the time of reperfusion as well as the cell type and the time of H/R were the key factors influencing the level of autophagy; In the study of MIRI, the type of animal, the time of ischemia rather than the time of reperfusion, cell type and time of hypoxia had a greater effect on the level of autophagy; In the study of RIRI, animal species as well as the time of ischemia were the key factors influencing the role shift of autophagy; In the study of HIRI, the choice of anesthetic (pentobarbital and ketamine), the time of ischemia and reperfusion had an effect on the level of autophagy; In the study of IIRI, the role of autophagy may be related to the duration of ischemia and animal species. An experiment investigating how aging affects autophagy levels revealed an age-dependent decrease in autophagy activity in adult *Caenorhabditis elegans* and confirmed that *daf-2* and *glp-1* mutants regulate autophagy in different spatiotemporal and spatial-specific ways to prolong the lifespan of *C elegans.*^[106]^ However, the effect of animal age on autophagy levels has not been observed in the aforementioned rodent studies, perhaps because of differences in basal autophagy levels and autophagy regulation patterns in different species. Surgery and trauma usually cause peripheral inflammation, which in turn can induce a pain hypersensitivity reaction characterized by reduced pain thresholds, increased pain intensity, and anomalous pain. The cytoplasmic clearance function of autophagy is by default anti-inflammatory in any type of cell.^[107,108]^ Therefore, in animal models of IRI, well-managed analgesia attenuates the inflammatory response and avoids excessive activation of autophagy. This seems to explain why experimental animals using anesthetics with well-established analgesia often exhibit moderate levels of autophagy. The findings for MIRI show that autophagy is activated in myocardial ischemia, but increases more during reperfusion.^[109]^ Moreover, autophagy during myocardial ischemia is protective and autophagy during reperfusion leads to cardiac injury.^[110]^ This is in disagreement with our observations, and further studies are needed to investigate the dynamics of autophagy levels during myocardial ischemia and reperfusion, and whether its role in damaged myocardium is a devil or an angel.

Autophagy is a dynamic process of IRI. A comprehensive understanding of the level of autophagy at different stages, the effects of autophagy on tissue damage and organ function, and the ability to assess the level of autophagy using simple laboratory assays are essential for strategies targeting autophagy to mitigate IRI in the clinical setting. Due to the limitations of current assays and clinical ethics, studies on autophagy in IRI are limited to the animal and cellular levels. Clinical data are urgently needed to verify the reliability of basic research results and the feasibility of mitigating organ IRI by targeting the autophagy level and to provide a theoretical basis for the development of individualized therapeutic regimens for patients with different organ IRI in clinical settings.

Additionally, autophagy does not operate in isolation in IRI and may influence cell outcomes postinjury through interactions with other forms of programmed cell death (PCD). Autophagy is activated under stress conditions such as amino acid deficiency, responses to unfolded proteins, or viral infections. Such stimuli can also activate other types of PCD and collectively determine the final cellular outcome. Studies have confirmed that the interactions between autophagy and various PCD modes play a role in the pathological process of IRI. For example, the interplay between autophagy activation and ferroptosis is involved in USP1 regulation of spinal cord IRI.^[98]^ Autophagy and apoptosis interact through calcium ion signal transduction and jointly regulate cell fate.^[102]^ Autophagy further limits inflammation by targeting pro-inflammatory signaling components.^[103]^ The impact of this crosstalk on IRI outcomes, whether other PCD types similarly exhibit a “double-edged sword” role in IRI progression, and the synchronous changes in these cell death modes require further investigation. This necessitates the exploration of multitargeted therapeutic approaches.

## Author contributions

**Conceptualization:** Guilin Zhou.

**Funding acquisition:** Wenya Bai, Jianlin Shao.

**Project administration:** Jianlin Shao.

**Resources:** Jia Liu, Jianlin Shao.

**Supervision:** Wenya Bai, Jia Liu, Junjie Li, Jianlin Shao.

**Visualization:** Lanlan Zhang.

**Writing – original draft:** Guilin Zhou.

**Writing – review & editing:** Guilin Zhou, Lanlan Zhang, Wenya Bai, Jia Liu, Junjie Li, Huan Jiang, Xin Li, Jianlin Shao.
